# Supplementary material for: A Bait-and-Hook Hydrogel for Net Tumor Cells to Enhance Chemotherapy and Mitigate Metastatic Dissemination
Source: Pharmaceutics. 2024 Nov 25;16(12):1516. doi: 10.3390/pharmaceutics16121516 (PMC11728792; doi:10.3390/pharmaceutics16121516)
Supplement: Supplementary file 1 [file pharmaceutics-16-01516-s001.zip › pharmaceutics-3251121-supplementary.pdf]

A bait-and-hook hydrogel net tumor cells to enhance chemotherapy and mitigate metastatic dissemination

Cailian Chen [1,†], Jinying Liu [1,†], Hongbo Zhang [2], Hongrui Zhang [1], Yanhui Liang [3], Qilian Ye [1], Wei Shen [1], Haibin Luo [1,*] and Ling Guo [1,*]

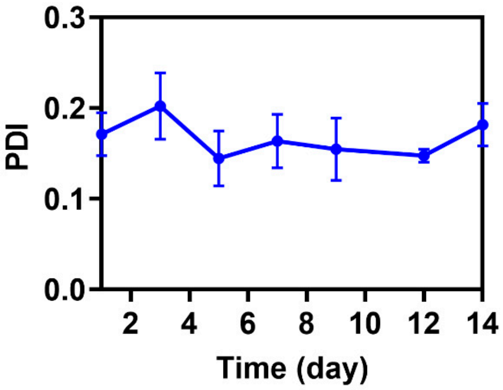

Figure S1 Polydispersity index of Dox Lipo for 14 days.

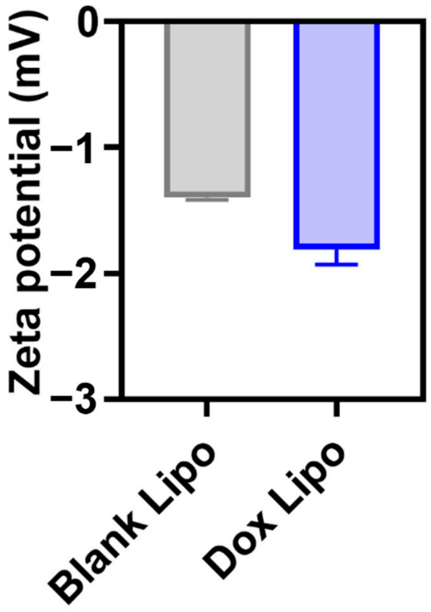

Figure S2 Zeta potential of Dox Lipo.

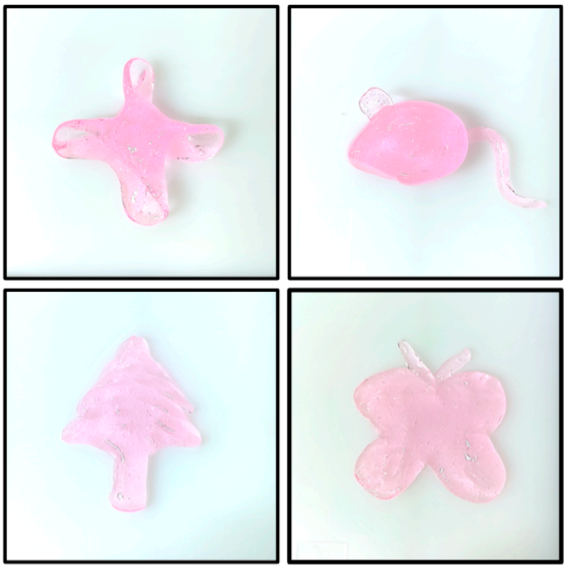

Figure S3 Optical images show the moldable features of BH-gel.

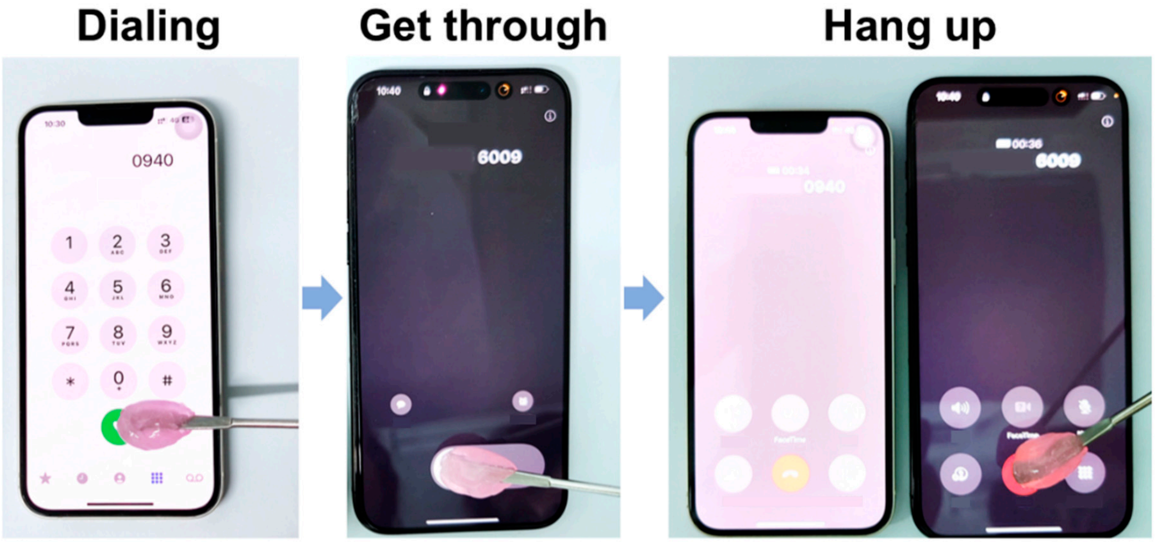

Figure S4 The electrical conductivity of BH-gel.

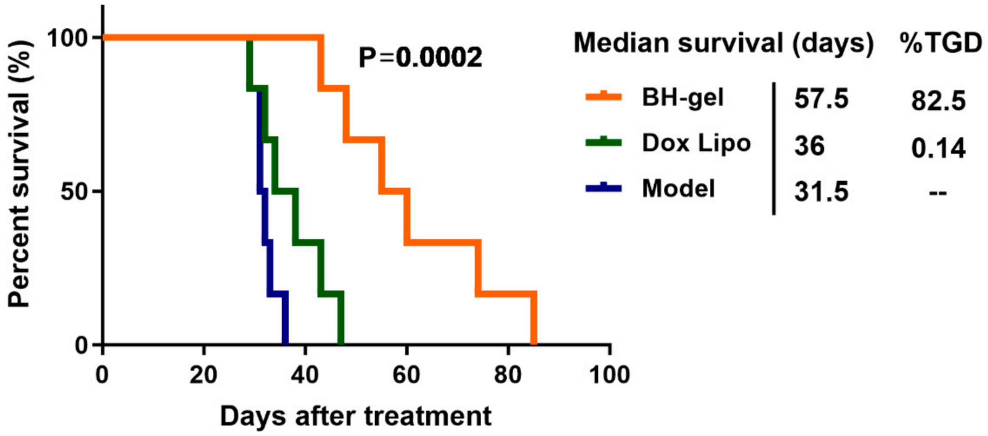

Figure S5 Kaplan−Meier survival curves and the median survival time of lung carcer-bearing mice after treatment. (n = 6 per group). Treated group (T) − Control group (C) = difference between median survival (days) of T vs. C (TGD). (T − C)/C (%TGD).
